# Convalescent plasma therapy for COVID-19 – Donor selection strategies and establishment of a plasma bank

**DOI:** 10.1016/j.nmni.2024.101525

**Published:** 2024-11-02

**Authors:** Mikael Kajova, Tamim Khawaja, Iris Levonen, Jukka-Pekka Pietilä, Jenni Virtanen, Sari H. Pakkanen, Hanna Välimaa, Arttu Nousiainen, Jussi Hepojoki, Tarja Sironen, Antti Vierikko, Jarkko Ihalainen, Olli Vapalahti, Anu Kantele

**Affiliations:** a Meilahti Vaccine Research Center, MeVac, Department of Infectious Diseases, University of Helsinki and Helsinki University Hospital, Helsinki, Finland; b Department of Infectious Diseases, Inflammation Center, University of Helsinki and Helsinki University Hospital, Helsinki, Finland; c Human Microbiome Research Program, University of Helsinki, Finland; d FIMAR, Finnish Multidisciplinary Center of Excellence in Antimicrobial Resistance Research, University of Helsinki, Finland; e Department of Virology, University of Helsinki, Helsinki, Finland; f Department of Veterinary Biosciences, Faculty of Veterinary Medicine, University of Helsinki, Helsinki, Finland; g Institute of Veterinary Pathology, Vetsuisse Faculty, University of Zürich, Zürich, Switzerland; h Finnish Red Cross Blood Service, Helsinki, Finland; i HUS Diagnostic Center, HUSLAB, Clinical Microbiology, Helsinki University Hospital, Helsinki, Finland

**Keywords:** COVID-19, SARS-CoV-2, Convalescent plasma, Plasma bank, Neutralising antibodies

## Abstract

**Background:**

Early in the COVID-19 pandemic, convalescent plasma (CP) emerged as a potentially effective treatment neutralising SARS-CoV-2. Early CP therapy with high neutralising antibody (NAb) titre may benefit COVID-19 outpatients and, in sufficient quantities even some hospitalised patients. This study details the process of setting up a CP bank, containing high- and low-titre CP for a clinical trial.

**Study design and methods:**

We identified 18–65-year-old convalescents with SARS-CoV-2 NAb titres of ≥1:40 in microneutralisation test (MNT). Following eligibility pre-screening, the Finnish Red Cross Blood Service (FRCBS) determined suitability as CP donors.

**Results:**

Of the 6466 COVID-19 convalescents contacted, 1481 provided serum, with 851 (57.5 %) exhibiting NAb titres ≥1:40. Participation barriers included reluctance, advanced age and, for women, insufficient body size. Of the volunteers, 125 were evaluated at FRCBS, with major exclusions for HLA antibodies (42 women), interferon antibodies (five men), and NAb titres waning below 1:20 (16 participants). Finally, 70 underwent plasmapheresis, resulting in 50 suitable CP donors (0.8 % of initial contacts and 3.4 % of those tested for NAb).

**Discussion:**

The process of setting up a CP bank proved challenging. Excessive laboratory workloads during a pandemic hamper their ability to conduct MNT, underscoring the need for rapid screening tests. Only a small proportion of our convalescents exhibited high-titre CP, this fraction declining over time because of waning immunity. Strict plasmapheresis criteria further constrained donor eligibility. Establishing a plasma bank requires meticulous planning to maximize efficiency. Detailed insights from current experiences may prove critical in future pandemics before other remedies and vaccines become available.

## 1 Introduction

The rapidly increasing number of patients with severe COVID-19 early in the pandemic, brought about an urgent yet unmet global need for effective treatments. In 2020, prior to the availability of vaccines or antivirals, convalescent plasma (CP), i.e. plasma from recovered patients appeared as a particularly appealing approach, given its expanding supply and potential to neutralise the SARS-CoV-2 virus. Initially, randomised controlled trials (RCTs) focused on hospitalised patients with severe COVID-19, revealing a lack of efficacy [1,2]. In later studies, CP given to newly diagnosed outpatients prevented progression of the disease to a moderate or severe form requiring hospitalisation [3,4]; however, conflicting results have also been reported [5,6]. High-titre CP was reported to benefit patients with severe disease when given in large doses [7]. Furthermore, CP retains a special value in treating immunocompromised patients [8].

Plasma donation is a prerequisite for any CP therapy. This entails not only donor recruitment, but also the establishment of a system for collecting, analysing, storing, and promptly delivering CP units to patients. Lack of resources for rapid assessment of NAb has led to practices in which CP with unknown NAb titres is given or, in many trials, enzyme-linked immunosorbent assay (ELISA) results have been used as a surrogate marker in lieu of performing a neutralisation assay [1,2,9].

The challenges of establishing a CP bank have been previously described [2,[10], [11], [12], [13], [14]]: for example, Harvala et al. [12] reported data on 436 donors and commented on the rapid decline of NAb levels and insufficient immune response in individuals with mild disease. We describe the multi-step process of assembling a bank of blood type-compatible plasma with predetermined NAb titres, aiming to portray the early pandemic scenario with no vaccines or remedies available. To our knowledge, this is the most detailed description of such a process up to now.

## 2 Methods

### 2.1 Study design and data collection

HUS Helsinki University Hospital (HUS) provides secondary and tertiary care for 1.7 million people. We describe here the process of establishing a plasma bank later used in a randomised, double-blinded clinical trial on CP [15]; for the purposes of the trial, our CP bank needed both high (≥1:160) and low (1:20–1:80) NAb titre CP units. Our volunteers were initially recruited to C_COVID master study (see below), which characterised COVID-19 symptoms and the associated immune response. It served as a screening process for plasma donors.

Ethics clearances were obtained from the Ethics Committee of HUS. Three studies – jointly referred to as C_COVID master study – covered the recruitment to the initial screening: Clin_COVID (HUS/1238/2020), Commun_COVID (HUS/1239/2020) and SARS-CoV-2/COVID-19 (HUS/853/2020) [16,17], and the fourth, Plasma_COVID-19 (HUS/1637/2020), covered the consenting to plasma donation and the clinical trial [15].

### 2.2 Recruitment, sampling, and questionnaires

Between May 1, 2020 and May 3, 2021, we identified in the HUS Infection database 33 437 adults with positive SARS-CoV-2 RT-PCR and invited each day 10–100 first adults tested to the C_COVID master study assessing NAb titres. All participants provided written informed consent; willingness to plasma donation was not a prerequisite for participation in NAb analyses. Blood samples were collected upon recruitment, and for some volunteers, repeated blood sampling was performed, to assess NAb kinetics and identify potential CP donors.

For the present study, we selected from C_COVID master study 18–65-year-old volunteers with NAb titres ≥1:40, seeking to ensure sufficient post-screening levels of NAb. To focus on the early pandemic stage, we excluded those vaccinated before recruitment. Serological data taken after vaccinations or reinfection are not presented in this report.

After preliminary screening, the potential donors were asked to fill in the standard Finnish Red Cross Blood Service (FRCBS) blood donor eligibility questionnaire. Those aged 60–65 were excluded unless they had donated blood before as per FRCBS guidelines. Starting December 2020, we prioritized males: they are more likely to meet the weight/height criteria for plasmapheresis.

### 2.3 Donor assessment at FRCBS

At the preliminary FRCBS visit, eligibility was confirmed according to blood donation guidelines [18,19]. Blood samples were analysed for standard blood safety parameters, for type I interferon alpha 2b (IFN-⍺2b) and HLA antibodies (all women, men who had received blood transfusions) [18,19]. Plasma was donated up to 3 times with a 14-day minimum interval.

### 2.4 Serological and virological methods

All participants had COVID-19 confirmed by a positive nasopharyngeal RT-PCR test [20,21]. The SARS-CoV-2 recombinant spike protein [22] was prepared for SARS-CoV-2 IgG enzyme-linked assay (ELISA) as previously described [23]. In the microneutralisation test (MNT), performed utilizing Wuhan-like D614G VeroE6-adapted C1P1 strain of SARS-CoV-2 [21,24], the sera were titrated starting from 1:20, with titres ≥1:20 considered positive. Donated plasma was studied with the same serological methods.

The IFN-α2b antibody ELISA (ImmunoTools, Friesoythe, Germany) has been described previously [25]. HLA antibodies were screened at FRCBS laboratory according to routine protocols by Luminex (Luminex Corporation, USA), One Lambda Labscreen kits and Fusion Software (Thermo Fisher Scientific, USA).

CP units with NAb ≥1:20 were included in the plasma bank. Regular protocol checks for donated plasma were made and archive samples collected according to European Directorate for the Quality of Medicines & Healthcare (EDQM) guidelines for fresh frozen plasma [19].

### 2.5 Statistics

Statistical analyses were performed in R version 4.3.2. The Wilson method was used to determine binomial confidence intervals using the “binom” package and figures were produced using the package “ggplot”. SPSS v. 27.0 (IBM Corp., Armonk, NY, USA) was used for binary logistic regression to analyse the association between sex or illness severity and findings of high-titre NAb, while chi-square/Fisher's exact test, as appropriate, were used to compare groups.

### 2.6 Data availability

To protect the anonymity of our participants, individual-level data are not provided. For any other relevant data, please contact the authors.

## 3 Results

### 3.1 Donor screening and recruitment

We invited 6466 SARS-CoV-2 RT-PCR-positive COVID-19 patients to the C_COVID master study which analysed NAb by MNT; samples were obtained from 1481/6466 (22.9 %) invitees aged 18–65 years, 57.5 % (851/1481) of them had NAb ≥1:40 and were considered potential donors.

**Fig. 1:**
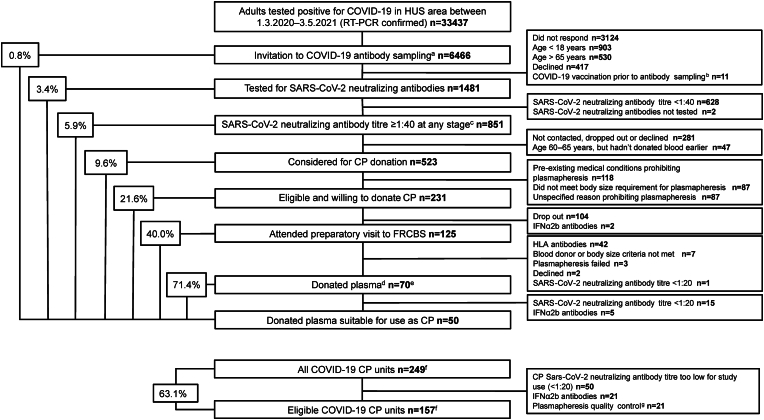
**Flow chart illustrating the recruitment process of COVID-19 convalescents for the initial serological study and subsequent participation as convalescent plasma (CP) donors. The figure includes details on excluded participants and the number of collected CP units.** Abbreviations: HUS = HUS Helsinki University Hospital, RT-PCR = reverse transcription-polymerase chain reaction, COVID-19 = Coronavirus Disease 2019, CP = convalescent plasma, FRCBS = Finnish Red Cross Blood Service, IFN-α2b = interferon alpha-2-beta, HLA = human leukocyte antigen ^a^ Volunteers invited amongst participants of serological/epidemiological studies, Clin_COVID, Commun_COVID and SARS-CoV-2/COVID-19. ^b^ COVID-19 vaccination was not an exclusion criterion for the serological/epidemiological studies. ^c^ Serology includes the highest titre from all samples of each patient, including titre rises due to reinfection or vaccination. It is thus higher than the number presented in the results section (799), which does not include samples collected after vaccination or suspected reinfection. ^d^ Each donation resulted in provision of three separate 200 mL CP units. CP could be donated multiple times with at least 60 days between occasions. ^e^ Eight vaccinated participants donated CP only after receiving COVID-19 vaccination. One of them donated once before and once after the vaccination had induced a rise in NAb levels. ^f^ Including 21 CP units from vaccinated participants. ^g^ FRCBS uses some donated plasma units for quality control as a routine practice.

Of the volunteers with NAb ≥1:40, 27.1 % (231/851) passed our preliminary screening; of them, 54.1 % (125/231) attended their FRCBS visit, where only 56.0 % (70/125) of potential donors met the more stringent blood donation criteria. Overall, after the exclusions, 70/1481 (4.7 %) of those initially screened for NAb donated plasma, i.e. 1.1 % (70/6466) of those initially invited to the C_COVID master study. Of the resulting 249 CP units, after exclusions (low NAb, IFN-⍺2b antibodies, quality control), 157 units (63.1 %) from 50 donors were finally suitable for the trial (Fig. 1).

### 3.2 Donor population

The donor demographics are presented separately for those recruited before and after refocusing to prioritize men (Table 1). After refocusing, the proportion of recruited men increased from 38.0 % to 87.9 %, and the recruitment success from 4.6 % (62/1349) to 6.1 % (8/132), p = 0.45.

**Table 1:** Characteristics of potential donors presented separately for those recruited before and after changing recruitment strategy to prioritising men.

	Before recruitment change n = 1349	After recruitment change n = 132	Donors^a^^b^ n = 70
**Donated,** n (%)	62 (4.6)	8 (6.1)	NA
**Female sex,** n (%)	837 (62.0)	16 (12.1)	19 (27.1)
**Age years,** median (IQR)	44 (34.0–55.0)	44 (33.0–52.0)	43.5 (34.3–54.0)
**BMI kg/m**^**2**^, median (IQR)^c^	25.5 (22.8–29.0)	25.8 (23.8–28.7)	27.8 (25.2–31.1)
**Weight,** median (IQR)^c^	76.0 (65.0–88.0)	83.5 (75.8–94.3)	90.0 (78.0–99.0)
**COVID-19 severity,** n (%)			
Outpatients	1189 (88.1)	118 (89.4)	52 (74.3)
Hospitalised	123 (9.1)	12 (9.1)	15 (21.4)
ICU	37 (2.7)	2 (1.5)	3 (4.3)
**Days from positive RT-PCR to**			
Study^d^ enrollment, median (IQR)	34.0 (18.0–185.0)	22.0 (17.0–30.0)	28.0 (17.0–127.8)
First blood sample, median (IQR)	43.0 (28.0–198.0)	31.0 (26.0–43.3)	34.0 (28.0–112.5)
First plasma donation, median (IQR)	150.5 (125.5–247.5)	133.0 (97.3–139.8)	146.0 (121.2–200.8)

a Out of 70 participants who donated plasma via plasmapheresis, it was eligible as CP for 50 individuals: plasma from 15 participants showed no measurable NAbs and five had interferon antibodies.

b Includes eight donors who underwent plasmapheresis only after vaccination and one who donated once before vaccination and once after.

c Data missing for 113 participants before recruitment strategy change, for 12 after the change, and for six of the donors.

d Serological/epidemiological studies titled Clin_COVID, Commun_COVID and SARS-CoV-2/COVID-19.

The mean time from positive RT-PCR to first plasma donation was 146 days. Some of the donations were made later, after a secondary NAb rise due to vaccination or re-infection (Fig. 1).

### 3.3 Donor exclusion

In multivariable analysis, high MNT levels were associated with hospitalisation but not with sex (Table 2).

**Table 2:** Proportion of participants^a^ with high neutralising antibody titres (≥1:160) 14–60 days after a positive SARS-CoV-2 RT-PCR test categorized by sex and severity of COVID-19.

	Total n	High titre^b^ n (%)	Low/negative titre^b^ n (%)	aOR (95 % CI)^c^	P-value
**Sex**					
Female	434	60 (13.8)	374 (86.2)	Ref.	Ref.
Male	348	58 (16.7)	290 (83.3)	1.2 (0.8–1.8)	0.383
**Hospitalisation**					
No	760	102 (13.4)	658 (86.6)	Ref.	Ref.
Yes	22	16 (72.7)	6 (27.3)	17.0 (6.5–44.4)	<0.001

a Participants aged 18–65 years. For the four participants with 2–3 samples were taken during this timeframe, the highest titre was included. Not all participants provided samples 1–60 days after testing positive, resulting in lower participant numbers than in other tables.

b High titre defined as ≥1:160, low/negative titre as ≤1:80.

c Adjusted odds ratio and 95 % confidence interval by binary logistic regression.

Although women (28.1 %; 853/3033) participated significantly more frequently than men (18.3 %; 628/3433, p < 0.001) to the initial antibody assessment, both sexes volunteered equally as CP donors after learning that they had sufficient antibody levels (62.2 %; 312/502 versus 60.5 %; 211/349, p = 0.62). However, of potential female donors 27.9 % (87/312) were excluded for insufficient body size and 13.5 % (42/312) due to HLA antibodies. No men were excluded for these reasons. Of the 220 volunteers analysed for IFN-⍺2b autoantibodies, 7 (3.2 %) tested positive.

Of those with sufficient NAb titres, 14.0 % (49/349) of men and 4.2 % (21/502) of women donated CP, p < 0.001) (Table 3).

**Table 3:** Reasons for exclusion of potential plasma donors at different stages among male and female COVID-19 convalescents^a^.

	All	Male	Female	p^b^
**Invitation to antibody sampling,** n (%)	6466	3433	3033	
Age >65	530 (8.2)	232 (6.8)	298 (9.8)	<0.001
Age <18	903 (14.0)	481 (14.0)	422 (13.9)	0.91
Declined	417 (6.4)	207 (6.0)	210 (6.9)	0.14
Did not respond	3124 (48.3)	1877 (54.7)	1247 (41.1)	<0.001
COVID-19 vaccination before sampling	11 (0.2)	8 (0.2)	3 (0.1)	0.19
**Tested for antibodies,** n (%)	1481 (22.9)	628 (18.3)	853 (28.1)	<0.001
Sars-CoV-2 MNT titre <1:40	628 (42.4)	279 (44.4)	349 (40.9)	0.18
Sars-CoV-2 MNT not performed	2 (0.1)	0 (0)	2 (0.2)	0.51
**MNT titre** ≥ **1:40,** n (%)^c^	851 (57.5)	349 (55.6)	502 (58.9)	0.21
Not contacted, dropped out or declined	281 (33.0)	118 (33.8)	163 (32.5)	0.68
60–65 years old, no previous blood donations	47 (5.5)	20 (5.7)	27 (5.4)	0.83
**Considered for CP donation,** n (%)	523 (61.5)	211 (60.5)	312 (62.2)	0.62
Medical condition prohibiting plasmapheresis	118 (22.6)	54 (25.6)	64 (20.5)	0.17
Body size requirements not met	87 (16.6)	0 (0)	87 (27.9)	<0.001
Unspecified reason	87 (16.6)	35 (16.6)	52 (16.7)	0.98
**Invited to FRCBS visit,** n (%)	231 (44.2)	122 (57.8)	109 (34.9)	<0.001
Drop out or declined	104 (45.0)	64 (52.5)	40 (36.7)	0.02
IFN-⍺2b antibodies detected	2 (0.9)	2 (1.6)	0 (0)	0.5
**Attended FRCBS preparatory visit,** n (%)	125 (54.1)	56 (45.9)	69 (63.3)	0.01
HLA autoantibodies	42 (33.6)	0 (0)	42 (60.9)	<0.001
Blood donor criteria not met	6 (4.8)	3 (5.4)	3 (4.3)	1
Plasmapheresis failed	3 (2.4)	2 (3.6)	1 (1.4)	0.587
Declined	2 (1.6)	1 (1.8)	1 (1.4)	1
Body size insufficient	1 (0.8)	0 (0)	1 (1.4)	1
MNT titre <1:20	1 (0.8)	1 (1.8)^d^	0 (0)	0.45
**Donated plasma**^e^**,** n (%)	70 (56.0)	49 (87.5)	21 (30.4)	<0.001
CP MNT titre <1:20	15 (21.4)	12 (24.5)	3 (14.3)	0.53
IFN-⍺2b antibodies detected	5 (7.1)	5 (10.2)	0 (0)	0.31
**Donated useable CP,** n (%)	50 (71.4)	33 (67.3)	18 (85.7)	0.11

Abbreviations: CP = convalescent plasma, FRCBS= Finnish Red Cross Blood Service, HLA = human leukocyte antigen, IFN-⍺2b = interferon alpha-2-beta, MNT = microneutralisation test.

^a^Percentages on the main rows represent proportion of individuals remaining from the earlier stage. Percentages on the indented rows represent the proportion of individuals excluded at this stage due to the criteria specified.

a Percentages on the main rows represent proportion of individuals remaining from the earlier stage. Percentages on the indented rows represent the proportion of individuals excluded at this stage due to the criteria specified.

b Significance of the difference between women and men being excluded at each stage.

c Serology includes the highest titre from all samples of each patient, including titre rises due to reinfection or vaccination, It is thus higher than the number presented in the results section (799), which does not include samples collected after vaccination or suspected reinfection.

d The MNT result of this individual was received between the FRCBS visit and the schedule plasmapheresis, which had to be cancelled because of the low titre.

e Five men and three women donated CP only after SARS-CoV-2 vaccination.

### 3.4 Neutralising antibody titres

**Fig. 2:**
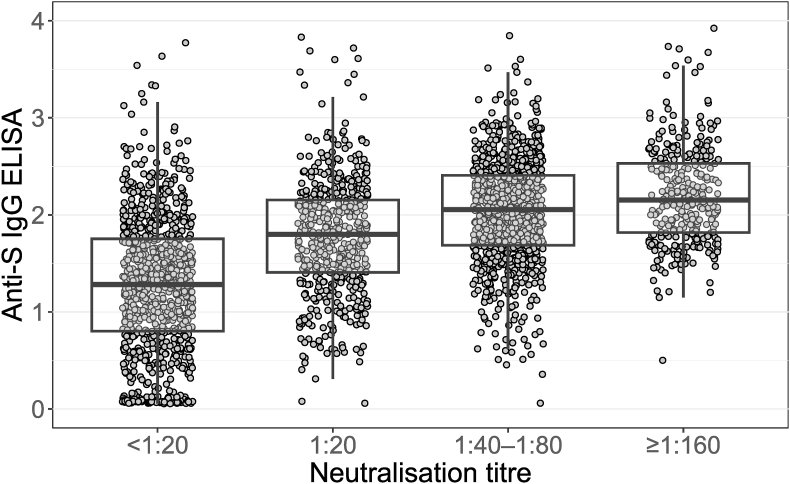
**Anti-S-IgG ELISA antibodies at various levels of anti-SARS-CoV-2 neutralising antibody titres as determined by microneutralisation test among COVID-19 convalescents (n = 1477)** A total of 2624 samples with available neutralising antibody (NAb) and ELISA results are presented. Samples were excluded if they were obtained after COVID-19 vaccination or after a suspected reinfection (i.e. late, significant rise of NAb titre). The four NAb groupings were established based on the requirements of our clinical trial [15]. For convalescent plasma, a NAb titre of ≥1:160 was considered high and a titre of 1:20–1:80 low. Anticipating waning of antibody levels, we only invited those with a titre of ≥1:40 to donate plasma.

**Fig. 3:**
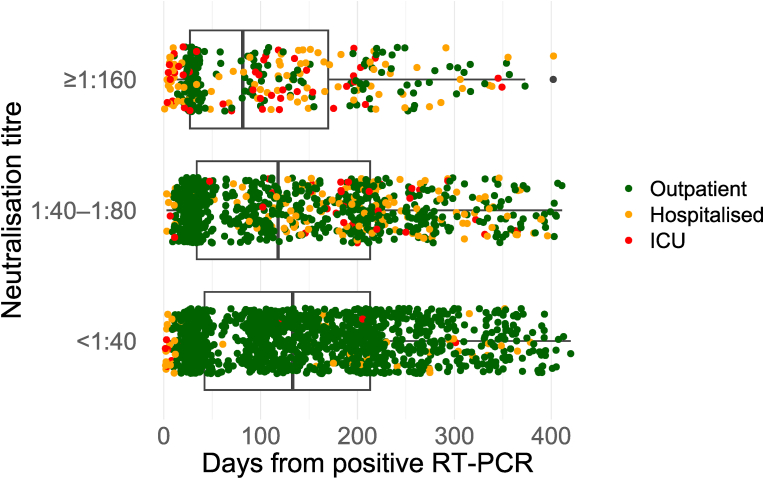
**Anti-SARS-CoV-2 neutralising antibody titre and time elapsed from positive RT-PCR test among COVID-19 convalescents as determined by a microneutralisation test**. The convalescents are categorized by disease severity into three categories: not hospitalised, hospitalised (non-ICU), and treated in an ICU. The figure includes pseudoreplicates: data from all analysed samples are presented (except for post-vaccination samples and those taken after suspected reinfection), encompassing multiple samples from same individuals obtained at different timepoints.

**Fig. 4:**
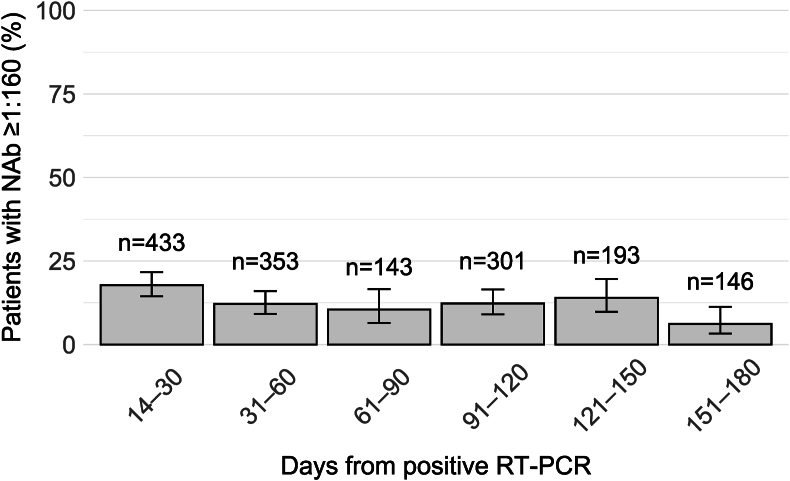
**The proportion of screened individuals with a high (≥ 1:160) titre of neutralising antibodies (NAb) against SARS-CoV-2 following RT-PCR-confirmed COVID-19 infection.** Whiskers represent binomial proportion 95 % confidence intervals as determined by the Wilson method. For participants who provided multiple samples during a given timeframe, the highest value was selected. Samples taken after vaccination or reinfection-induced rise in antibody titres are not included. The data comprises 1569 analysed samples from 1013 participants.

Of all 3301 blood samples collected from our 1481 volunteers, we excluded from final data the following: 304 samples drawn after a COVID-19 vaccination; 103 taken after presumed reinfections (at least 4-fold rises in NAb titres); 264 lacking NAb results. Fig. 2, Fig. 3, Fig. 4 and Table 2 are based on 2630 samples analysed for NAb drawn from 1478 participants (one was vaccinated before the study, and for two, NAb were not analysed). Of these 1478 individuals, 799 (54 %) had NAb ≥1:40 and 233 (16 %) a high titre (≥1:160) at some point during the study.

Overall, the proportion of convalescents with high-titre plasma was low: 18 % at 14–30 days and 6 % at 151–180 days (Fig. 4). Plasma with low NAb titres, qualifying as LCP (1:20–1:80) (Fig. 3, Fig. 4), was obtained even over six months after the initial COVID-19 episode (Table 1). The ELISA and MNT results did not correlate well (Fig. 2).

## 4 Discussion

Although not known during our clinical CP trial planning, later studies have indicated that only high-titre CP may be efficacious [8,26]. We present a 14-month process of recruiting COVID-19 convalescents as plasma donors and establishing a CP bank, which finally comprised 157 units of CP with predetermined NAb titres from 50 individuals. Although theoretically straightforward, we found the process to be highly inefficient: only 0.8 % of individuals invited to preliminary evaluation (serology testing) and 5.9 % of those with NAb ≥1:40 in pre-screening finally donated plasma.

### 4.1 Key points for creating a CP bank

We identified two key requirements for recruiting CP donors: sufficient NAb titres and eligibility for plasmapheresis. Below, we discuss the approaches related to meeting these two criteria.

Recruitment should concentrate on convalescents most likely to have high CP titres. However, only 16 % of our participants had a NAb titre of ≥1:160 at any stage (excluding samples taken after vaccination or suspected reinfection), concurring with the rate of 25.9 % with high antibody levels reported by Carter et al. [10]. This raises three points that warrant discussion.

Our data demonstrated higher titres for inpatients than outpatients, the finding concurring with many previous studies [[27], [28], [29]]. Klein et al. and Mehew et al. reported three factors predicting a high NAb titre: male sex, older age and severe COVID-19 requiring hospitalisation [27,29]. In our analysis, consistent with the report by Steenhuis et al. [30], sex did not independently predict a high NAb among those hospitalised for COVID-19.

A second aspect of concern is the waning of NAb levels within a few months – a phenomenon demonstrated in the present and numerous earlier datasets [12], [28], [31]. Indeed, Gontu et al. identified a window of only 60 days for collecting high-titre plasma [31]. We observed a similar trend: 18 % of participants had high-titre plasma at 14–30 days compared to 6 % at 151–180 days.

Emerging virus variants pose the third challenge. Patients may receive CP with NAb targeting an earlier variant, resulting in reduced virus neutralisation against the new variant infecting the patient. This underscores the importance of collecting CP with a high NAb titre, as it is more likely to exhibit cross-reactivity against other variants than low-titre CP [[32], [33], [34]]. To better match the virus variants, it seems epidemiologically logical to invite recently infected donors from the same geographical area as the recipients [8,26]. In accord with this, in our double-blinded randomised CP trial, we conducted an additional analysis by matching variant-specific CP, the data suggesting potential benefits of such an approach [15]. Hybrid immunity, achieved through vaccination after infection – or vice versa – may present a partial solution to the challenges outlined above. Vaccination boosts NAb titres, and higher titre CP is more likely also to have higher levels of antibodies cross-reactive with the new variant strains [[35], [36], [37]]. However, our study focused on establishing a CP bank early in a pandemic before vaccines have become available.

### 4.2 Meeting eligibility criteria of plasmapheresis

To optimise CP collection, we started focusing on those recently hospitalised and prioritized male participants, as they mostly do not need HLA antibody testing [18] and, importantly, often have higher weight, better meeting the criteria for plasmapheresis. However, the proportion of successful donor recruitments saw only a non-significant improvement (from 4.6 % to 6.1 %, p = 0.45). Collecting CP with a high NAb titre should not exclusively target recently hospitalised patients: not all of them are suitable donors, since many are elderly and/or multimorbid.

### 4.3 Determination of NAb levels of CP

A major challenge in establishing the CP bank was the delay in obtaining laboratory results, attributed to the excessive workload in laboratories due to the pandemic and to the labour-intensive nature of MNT requiring biosafety level 3 (BSL-3) conditions [11].

To expedite the process, we attempted pre-screening with a faster IgG-specific ELISA assay, but its results did not correlate well enough to identify individuals with high NAb titres. This finding contrasts with the results of some earlier studies [2,12,38]. The fact that ELISA was conducted at a single dilution (1:50), while a serial dilution of each plasma was studied in MNT, likely explains the observed discrepancy between the tests. Indeed, in many trials, such as the RECOVERY trial, Euroimmun IgG ELISA was used as a surrogate assay for estimating NAb titres [9], and another study reported successful screening with semiquantitative testing of nucleocapsid IgG [10]. Comparative studies indicate large variations in the performance of commercial ELISAs for detecting high NAb titres [34,39,40]. There is an obvious need for rapid, high-throughput proxy assays for neutralisation, such as those described by Rusanen et al. [23]. Since the early stages of the pandemic, vesicular stomatitis virus- and lentivirus-based pseudovirus assays for quantifying neutralising antibodies have been developed [41,42], their advantages including the rapid set up of neutralisation test for novel variants, exemption from BSL-3 conditions and generally good correlation with MNT [43]. Indeed, in lack of a rapid screening test, we missed many potential donors as their NAb titres had already waned below 1:20 before donation.

### 4.4 Previous data on how to create a CP bank

Some earlier studies, such as those from India, Italy, and USA have described the process of recruiting CP donors and establishment of a plasma bank during the COVID-19 pandemic [10,11,13,14]. However, despite similar challenges, the differences in approaches, assays and cut-offs in NAb titres, and country-specific factors hinder direct comparisons of efficacity [10,11,13,14]. In the US study by Carter et al., 6.8 % (209/3093) of respondents to an online survey qualified as donors [10]. In another US study only 0.2 % of contacted convalescents and 9.7 % of those responding to advertisements ultimately donated plasma [14]. The Indian study reported that 9.0 % of contacted convalescents finally donated plasma [13]. However, apart from the Italian study, NAbs were not determined. Even in the Italian study, they were systemically determined only from those donating plasma, not during the screening process.

### 4.5 Autoantibodies detected in CP

Type 1 interferon autoantibodies have been proposed as a mechanism for COVID-19 disease progression [44], although the risk appears negligible [45]. We screened our donors: 3.2 % (7/220) tested positive leading to exclusions. The proportion of anti-IFN-⍺2 autoantibodies accords with an earlier study [45].

### 4.6 Is convalescent plasma therapy still relevant?

The threat of the SARS-CoV-2 pandemic has lessened due to virus evolution, population immunity, and availability of vaccines and treatments. Due to the multitude of other therapies and the limited efficacy shown in RCTs, current guidelines recommend CP for COVID-19 only in high-risk ambulatory patients unable to receive antivirals [46]. However, knowledge gained from establishing CP banks for COVID-19 provides a foundation for future emerging viral outbreaks, ensuring we are not starting from scratch if CP therapy is needed before vaccines and antivirals are available. Setting NAb levels in advance has clear advantages, but a smooth process can only be achieved, if a rapid antibody test is used during the inital screening stage.

### 4.7 Strengths and limitations

The clear strength of our study is that it describes a real-life process amidst a pandemic before vaccinations become available. It describes in detail the process of establishing a plasma bank and the multitude of restrictions that finally translate into an ineffective process. Scrutinizing these individual steps reveals points of particular concern and thus helps future studies.

Some limitations deserve discussion. Our final proportions of donors may not directly indicate the yields from any convalescent population. By contrast, as it became necessary to enhance the selection process (see discussion above), our final proportions can be presumed to be slightly higher than the yields without any focusing. On the other hand, given the waning of NAb levels, it is plausible that we lost potential donors due to delays in obtaining NAb results.

## 5 Conclusions

Our major finding was that of the restricted proportion of convalescents willing to donate, only a minority meet the strict eligibility criteria of plasmapheresis. While NAb screening of all potential donors raises the quality of the CP bank, laboratory capacity and lack of a high throughput surrogate assay easily become a bottleneck in the process due to the urgency caused by waning NAb titres. A further challenge is set by the emergence of new virus variants: CP elicited by previous variants may not neutralise new variants effectively. As the odds of obtaining CP with matching virus type is increased for CP collected in the same area and time period [8,26], a local and constantly updated plasma bank, like ours, may be necessary.

## CRediT authorship contribution statement

**Mikael Kajova:** Formal analysis, Investigation, Writing – original draft, Writing – review & editing, Project administration. **Tamim Khawaja:** Validation, Formal analysis, Investigation, Data Curation, Writing – original draft, Writing – review & editing, Project administration. **Iris Levonen:** Formal analysis, Investigation, Data Curation, Writing – review & editing, Visualization, Project administration. **Jukka -Pekka Pietilä:** Formal analysis, Investigation, Data Curation, Writing – review & editing, Visualization. **Jenni Virtanen:** Investigation, Writing – review & editing, Visualization. **Sari H. Pakkanen:** Writing – review & editing, Project administration. **Hanna Välimaa:** Investigation, Writing – review & editing, Funding acquisition. **Arttu Nousiainen:** Investigation, Writing – review & editing. **Jussi Hepojoki:** Methodology, Investigation, Writing – review & editing. **Tarja Sironen:** Investigation, Writing – review & editing, Supervision. **Antti Vierikko:** Investigation, Resources, Writing – review & editing. **Jarkko Ihalainen:** Investigation, Resources, Writing – review & editing. **Olli Vapalahti:** Methodology, Resources, Writing – review & editing, Supervision, Funding acquisition. **Anu Kantele:** Conceptualization, Writing – original draft, Writing – review & editing, Supervision, Project administration, Funding acquisition.

## Funding

The trial was funded by the Finnish Government Subsidy for Health Science Research (TYH 2021315, TYH2023238), the Finnish Medical Foundation and the Academy of Finland (grant numbers 335527 and 336439).

## Declaration of competing interest

The authors declare that they have no known competing financial interests or personal relationships that could have appeared to influence the work reported in this paper.
